# Supplementary figures and images for: Baleen whale inhalation variability revealed using animal-borne video tags
Source: PeerJ. 2022 Jul 20;10:e13724. doi: 10.7717/peerj.13724 (PMC9308462; doi:10.7717/peerj.13724)

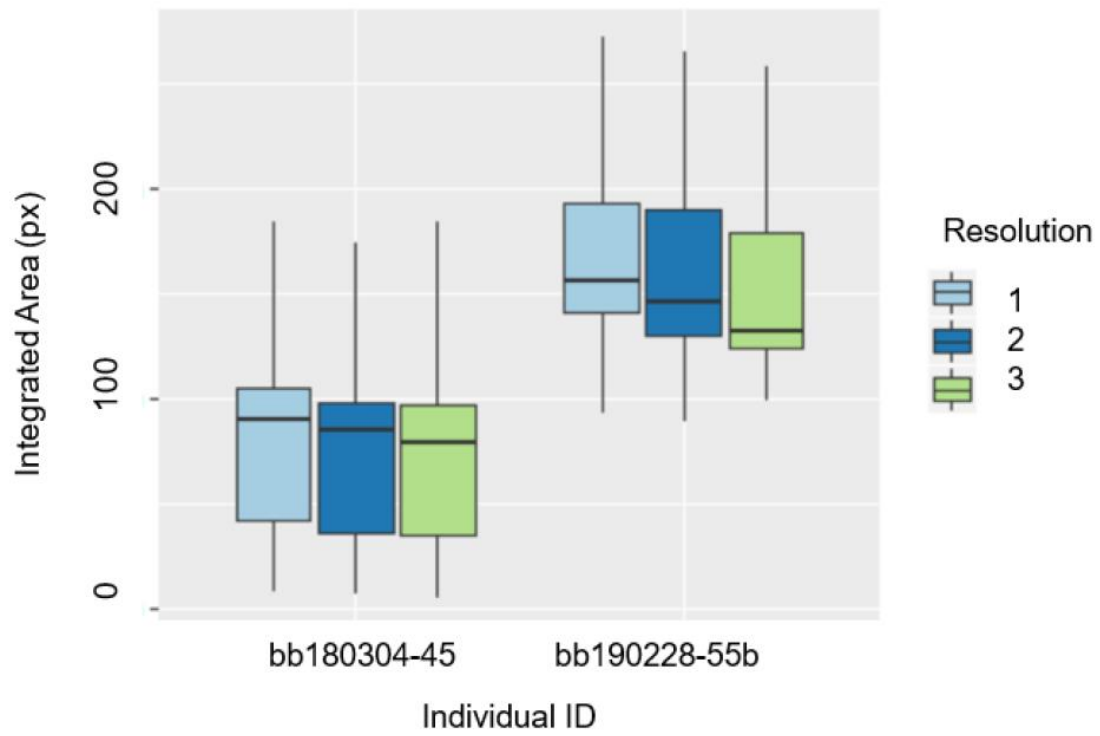

Supplement: Supplemental Information 1 — Boxplot showing the IA for a resolution of 1, 2, or 3 for two Antarctic minke whales. Resolution of 1 represents IA measurements which were calculated using every frame of the inhalation. Resolution of 2 represents IA measurements which were calculated using every 2 frames of the inhalation. Resolution 3 represents IA measurements using every 3 frames of an inhalation. Boxplot shows mean, standard error, maximum, and minimum. [file peerj-10-13724-s001.pdf]

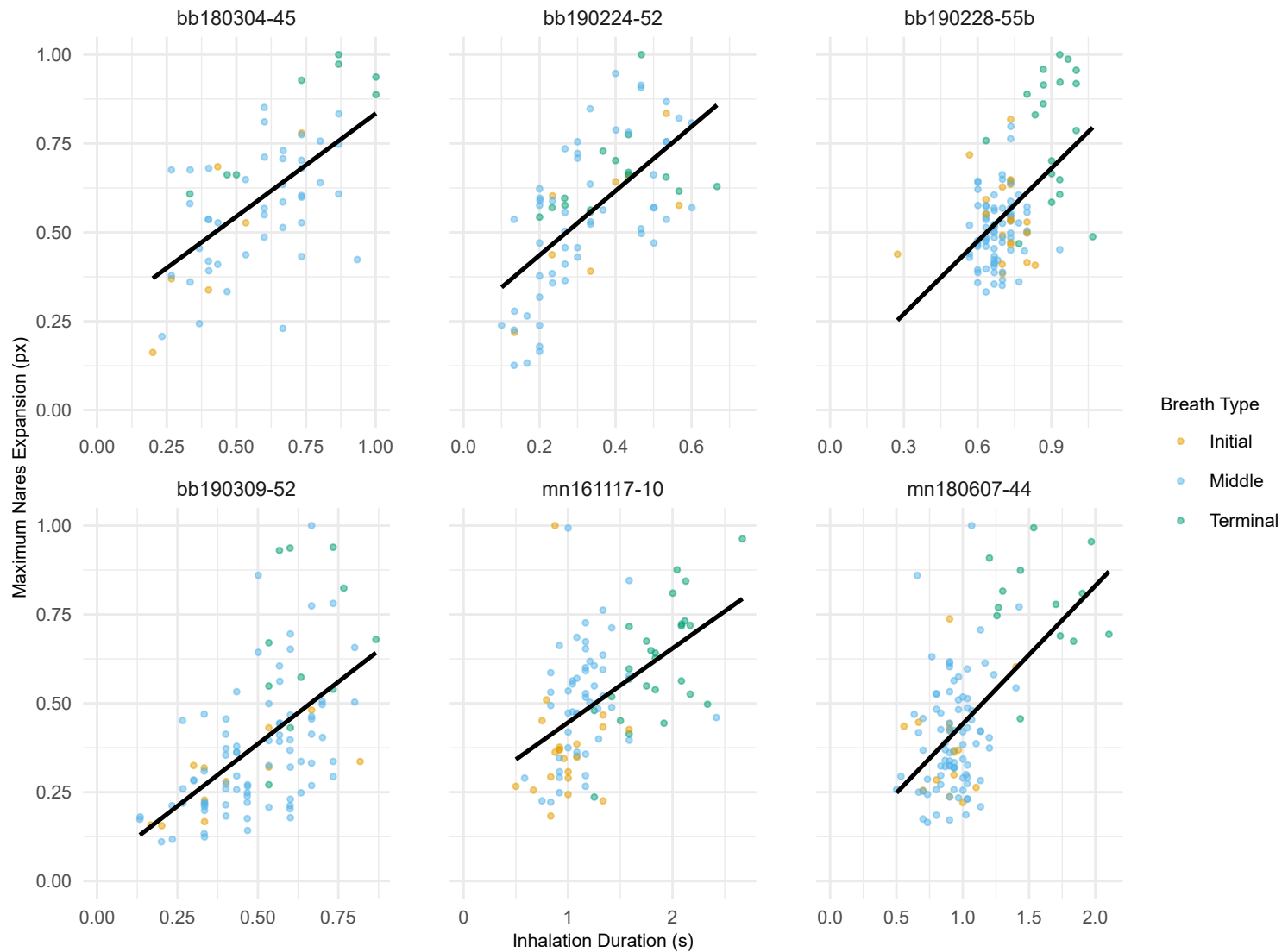

Supplement: Supplemental Information 2 — Normalized maximum nares area plotted against inhalation duration for all whales in the study. The black line represents the trend line. Observations were grouped by breath type (initial, middle, or terminal) and separated by whale field ID. The labels for each panel represent the species (mn = humpback, bb = Antarctic minke whale) and the field identification number. The y-axis was normalized to the largest IA for each animal. [file peerj-10-13724-s002.pdf]

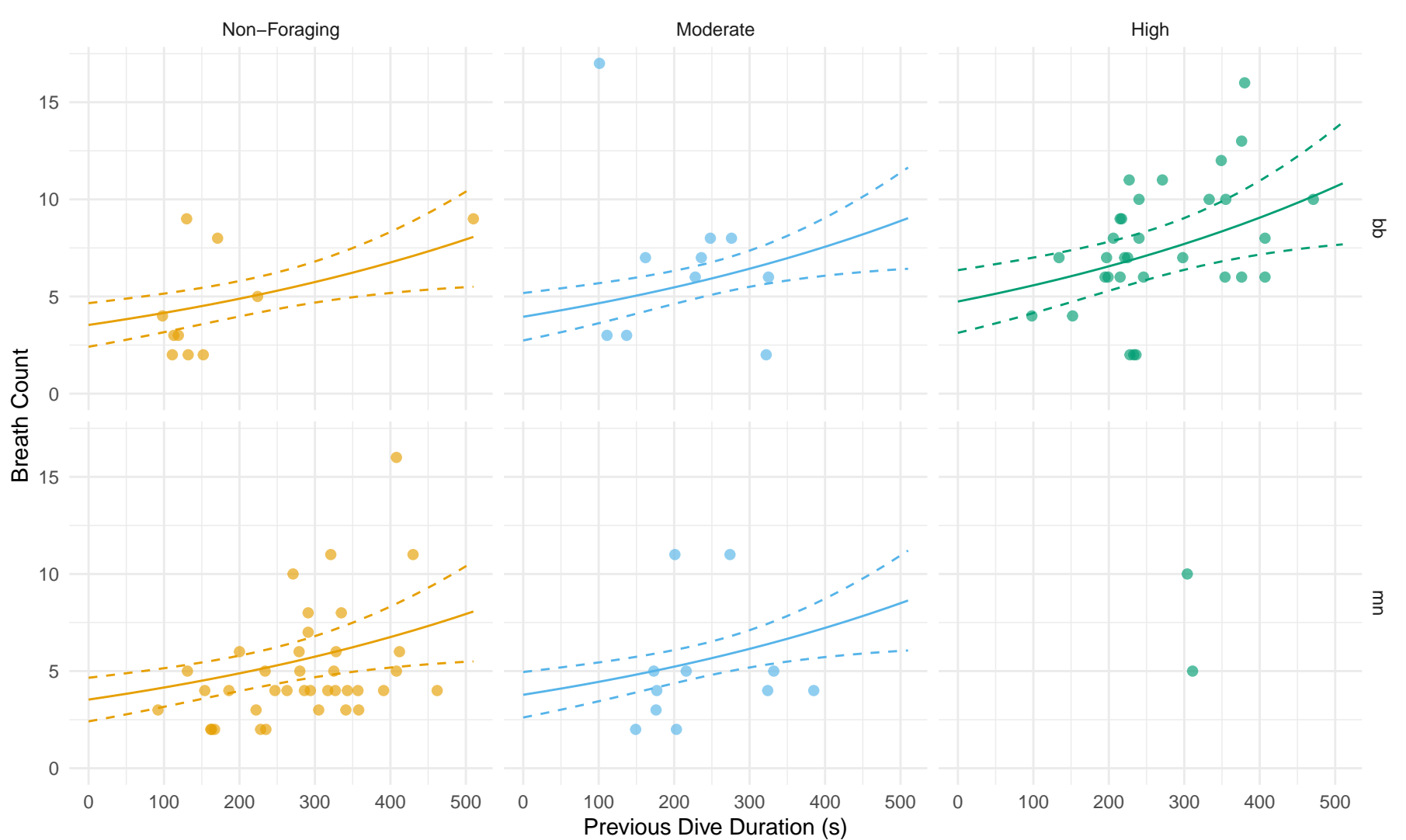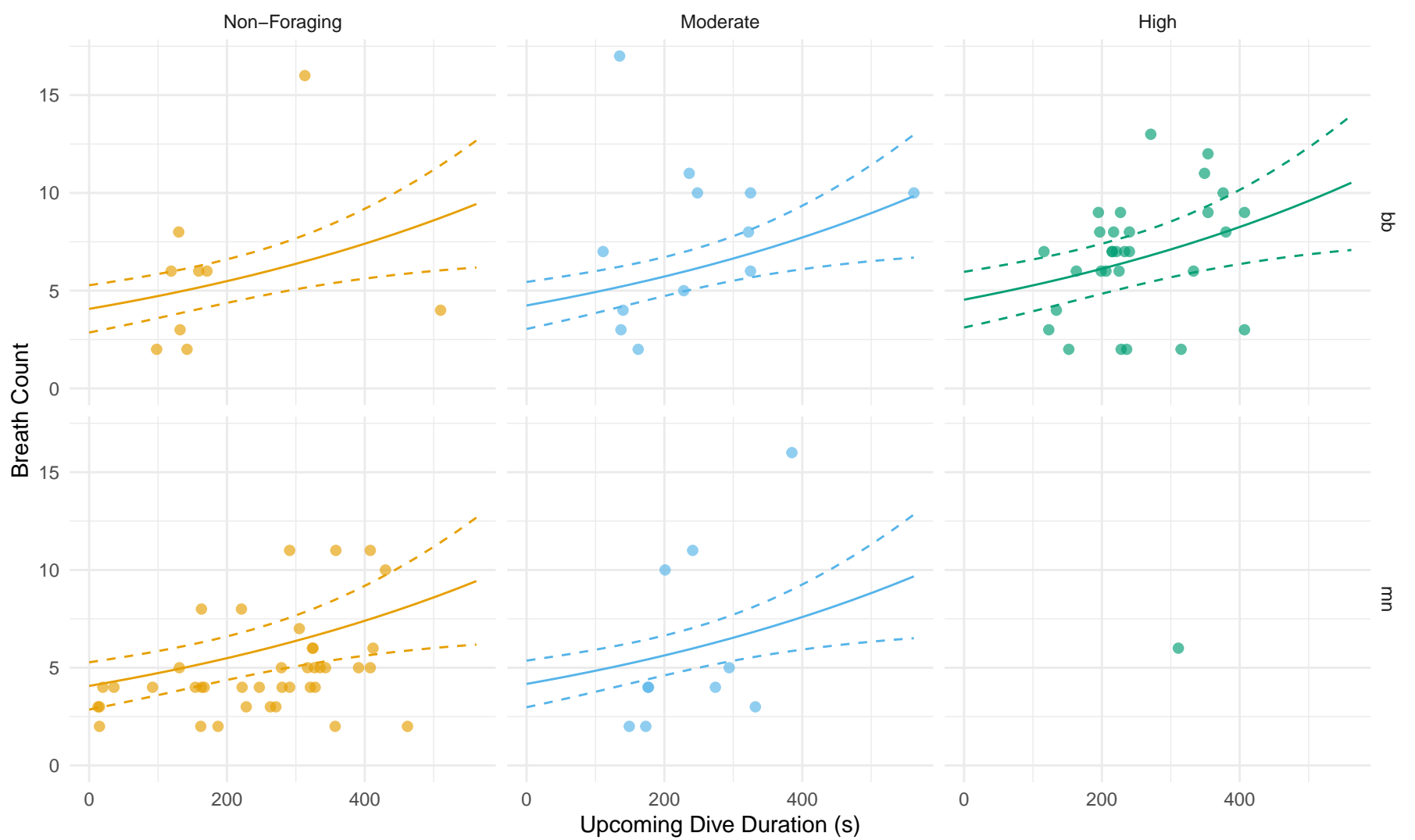

Supplement: Supplemental Information 3 — The breath count surface interval plotted against the upcoming dive duration. The colored lines are separated lunge count category defined separately for humpback and minke whales. For minke whales: non-foraging = 0 lunges; moderate = 1–4 lunges; high = g5 lunges. For humpback whales: non-foraging = 0 lunges; moderate = 1–2 lunges; high = g3 lunges. No model was plotted for high lunges for humpback whales due to small sample size. The lines represent the first degree polynomial GLMM where both lunge count and dive duration of the previous and upcoming dive were predictor variables, breath count was the response, and whale ID and species were random effects. Observations were grouped by species. The labels for each panel represent the species (mn = humpback, bb = Antarctic minke whale). [file peerj-10-13724-s003.pdf]
