# Supplementary material for: Baleen whale inhalation variability revealed using animal-borne video tags
Source: PeerJ. 2022 Jul 20;10:e13724. doi: 10.7717/peerj.13724 (PMC9308462; doi:10.7717/peerj.13724)
Supplement: Supplemental Information 4 — Statistics for model fit (AIC scores and log likelihood), fixed effects inference (p-value), effect size, and confidence intervals for each GLMM fit in this study. Effect size were calculated using the slope at the model’s median. Confidence intervals were calculated using the Wald method. [file peerj-10-13724-s004.docx]

| **GLMM Summary Statistics** | | | | | |
| --- | --- | --- | --- | --- | --- |
| GLMM Variables | AIC | Log Likelihood | *p-value* | Effect Size | 95% CI |
| Inhalation Duration ~ Breath Type | -254.0 | 132.0 | < 0.0001 | NA | Initial (0.414 to 0.835)  Middle (0.443 to 0.875)  Terminal (0.640 to 1.276) |
| IA ~ Breath Type | -353.5 | 181.8 | < 0.0001 | NA | Initial (0.222 to 0.329)  Middle (0.265 to 0.352)  Terminal(0.545 to 0.669) |
| Maximum Nares Area ~ Breath Type | -290.3 | 150.2 | < 0.0001 | NA | Initial (0.398 to 0.525)  Middle (0.436 to 0.538)  Terminal(0.690 to 0.788) |
| Maximum Nares Area ~ Inhalation Duration | -271.5 | 140.8 | <0.0001 | bb (0.880)  mn (0.450) | 1.281 to 1.878 |
| Total Inhalation Duration ~ Previous Dive Duration | 456.4 | -222.2 | 0.456 | 0.0181 | -1.651 to 0.741 |
| Total Inhalation Duration ~ Previous Dive Lunge Count | 461.4 | -224.7 | 0.604 | 0.912 | -1.242 to 0.722 |
| Total Inhalation Duration ~ Upcoming Dive Duration | 443.3 | -215.7 | 0.0024 | 0.0298 | -2.341 to -0.503 |
| Total Inhalation Duration ~ Upcoming Lunge Count | 453.6 | -220.8 | 0.00073 | 0.957 | -2.828 to -0.751 |
| Total Inhalation Duration ~ Upcoming Dive Duration and Lunge Count | 437.9 | -211.0 | Duration (0.0063)  Lunges (0.0010) | 0.0241 | Duration (-2.213 to -0.364)  Lunges(-2.614 to 0.659) |
| Breath Count ~ Previous Dive Duration and Lunge Count | 504.5 | -244.2 | Duration (0.610)  Lunges(0.161) | 0.0251 | Duration (-0.713 to 1.215)  Lunges(-1.4361 to 0.240) |
| Breath Count ~ Upcoming Dive Duration and Lunge Count | 502 | -243 | Duration (0.101)  Lunges(0.318) | 0.0310 | Duration (-1.809 to 0.162)  Lunges(-2.120 to -0.272) |
